# Workers’ Behavior and Hygienic Parameters Related to Blood Lead Levels: A Cross-Sectional Biomonitoring Study in an Italian Automotive Plant

**DOI:** 10.3390/toxics14100922

**Published:** 2026-10-18

**Authors:** Loreta Tobia, Riccardo Mastrantonio, Mario Muselli, Giorgia Nobili, Danilo Cottica, Vincenza Cofini, Stefano Necozione, Leila Fabiani

**Affiliations:** 1 Department of Life, Health and Environmental Sciences, University of L’Aquila, 67100 L’Aquila, Italy; loreta.tobia@univaq.it (L.T.); mario.muselli@univaq.it (M.M.); vincenza.cofini@univaq.it (V.C.); stefano.necozione@univaq.it (S.N.); leila.fabiani@univaq.it (L.F.); 2 Department of Biomedicine and Prevention, University of Rome Tor Vergata, Viale Montpellier 1, 00133 Rome, Italy; giorgia.nobili@ptvonline.it; 3 Environmental Research Centre, Istituti Clinici Scientifici Maugeri S.p.A. S.B., Via Atene 935010, Perarolo di Vigonza, 27100 Padova, Italy; danilo.cottica@icsmaugeri.it

**Keywords:** lead, occupational, exposure, biomonitoring, zinc-protoporphyrin, blood, hygiene, behavior, lifestyle, automotive

## Abstract

Occupational lead exposure is a relevant health concern, but the relationships between lead exposure, biomarkers, and individual, occupational, and hygienic characteristics remain incompletely understood. This study investigated associations between blood lead (Pb) and zinc-protoporphyrin (ZnPP) levels and occupational, clinical, individual, lifestyle, and hygiene-related characteristics in 464 exposed workers. Occupational and individual data were collected and statistically analyzed to identify associations between biomarkers and categorical or continuous variables. Blue-collar and maintenance workers showed significantly higher blood Pb levels than white-collar workers (11.07 and 10.42 vs. 4.81 µg/dL). Unadjusted analyses showed higher blood Pb levels among forklift users and workers in production units 1 and 2. No statistically significant differences in ZnPP were observed across the occupational categories examined. Female sex, non-smoking status, and longer hair were associated with lower Pb levels. Exploratory multivariable analyses identified associations of ZnPP with hypertension and physical activity, but the model explained only 3.65% of its variance. As expected, due to the low concentrations of xenobiotics detected, a weak association was observed between blood Pb and ZnPP. Overall, the findings suggest modest associations between biomarker levels and select individual/occupational characteristics (such as hair length and forklift use). Given the low proportion of explained variance, these exploratory observations should be interpreted with caution and warrant further research.

## 1. Introduction

Lead is a widely distributed industrial metal, and it is also naturally present in the environment. It is an environmentally persistent element, a major global hazard and it may cause work-related illness [1]. Lead contamination mainly occurs through absorption via the respiratory and gastrointestinal systems. In adults, lead is mostly accumulated in bones (up to 94%), with a mean half-life of 1 to 2 decades [2]. Percutaneous absorption of lead compounds may represent a relevant route of exposure, also in occupational settings [3]. The effects of lead exposure can involve both the generation of reactive oxygen species (ROS) and a direct depletion of antioxidant reserves [4]. Lead is also known to interfere with hemoglobin synthesis [5] and to increase the risk of abortion [2]. The nervous system may be affected by lead-related damage, both in young and adult patients, and moderate levels of exposure can impair brain development in children, causing cognitive dysfunction, hyperactivity and behavioral problems [6]. Lead is linked to an increased risk of abortion, and it has been associated with low birth weight of infants. In females, occupational exposure resulting in blood lead (PbB) levels > 10 µg/dL is associated with an increased risk of spontaneous abortion, premature delivery and low birth weight [2]. A systematic review conducted in 2024 [7] reported a variety of adverse fetal and neonatal outcomes, even at low blood lead levels. Effects on neurodevelopment and cognitive-behavioral deficits have been demonstrated. In men, lead exposure is linked to low libido, low semen volume and sperm counts, increased abnormal sperm morphology and decreased sperm motility, leading to impairment of reproductive function [2]. A recent review [8] linked Pb exposure to several negative effects on male fertility: low semen volume, sperm concentration, total sperm count, sperm vitality and sperm motility.

Total lead levels in body fluids or tissues, such as blood, bone, saliva, or urine, are assessed as exposure biomarkers for lead exposure. PbB is the most widely used, and it is considered to be the most reliable biomarker for general clinical use and public health surveillance [2]. Pb in bone is considered a biomarker of cumulative exposure, accumulating in both trabecular and cortical bone [9]. Tooth Pb is a potential indicator for long-term exposure (e.g., years) [10]. Saliva reflects recent lead exposure and may be used as an additional biomonitoring measure [11,12]. Hair has been explored as a noninvasive approach to estimate lead body burden [9]. Biomarkers of effect are defined as any measurable biochemical, physiological or other alteration within an organism that can be recognized as an established or potential health impairment or disease [2]; regarding lead exposure biomonitoring, zinc-protoporphyrin is a well-established biomarker [13].

The European Parliament set a blood lead reference value for occupational exposure of 30 µg/dL, and it will be further reduced to 15 µg/dL beginning in 2029, as per EU Directive 2024/869 [14]. Health surveillance is mandatory for PbB levels higher than 9 µg/dL, while additional restrictions are applied to female workers in childbearing age with PbB levels higher than 4.5 µg/dL [15]. The body burden of lead and its pathophysiological effects in occupationally exposed groups may be influenced by several factors, such as the regulatory framework for worker protection and the proposed exposure limit values, general safety measures, personal protective equipment and the occupational sector [16].

Based on what is stated so far, the present study aimed to highlight possible occupational, individual/clinical and lifestyle/hygienic parameters that are associated with blood lead and blood zinc-protoporphyrin levels. Although this is an observational study, the results may highlight associations that to date have not yet been explored. Future experimental research could confirm the observed relationships and determine their magnitude and relevance. To the best of our knowledge, a comparable study accounting at the same time for occupational, individual and hygienic characteristics is not available to date. Our findings may shed light on the relationship between exposure, effects and individual characteristics, with the aim of enhancing the protection of exposed workers.

## 2. Materials and Methods

This cross-sectional observational study enrolled workers from a lead-acid battery manufacturing plant located in Avezzano, Italy, to explore associations between occupational, clinical, and lifestyle/hygienic characteristics and exposure and effect blood biomarkers of lead monitoring. Data were obtained from clinical examinations and medical history records collected as part of routine occupational health surveillance conducted between July 2024 and February 2025. Written informed consent was obtained from all participants. The study was authorized by the Ethics Committee of the Abruzzo Region, Italy (CETRA, report No. 26, 4 July 2024). Overall, 464 workers were included, corresponding to a participation rate of 98.3% of the eligible workforce.

For each worker, venous blood samples were collected from the cubital vein using EDTA heparinized evacuated lead-free tubes (BD, Plymouth, UK). Blood specimens for evaluating both markers were obtained from the same phlebotomy procedure, in a fasting state, early in the morning, and at the start of the work shift. Biomonitoring activities were not conducted on workers returning from leave. Company closure periods did not interfere with the analyses, as the company operates continuously without production shutdowns. Blood lead levels were measured by Inductively Coupled Plasma–Mass Spectrometry (ICP–MS) [17] and expressed as µg/dL. The method allows a very low Limit of Detection (<10–12 ppt), based on a 98% confidence level. Zinc protoporphyrin (ZnPP) levels were measured by High-Performance Liquid Chromatography coupled with Fluorescence Detection (HPLC-FL) [18] and were also expressed as µg/dL in whole blood. The method has a Limit of Detection of 0.2 µg/dL and a Limit of Quantification of 0.5 µg/dL (Eureka Lab Division, AN, Italy). The reference range for ZnPP is 5–40 µg/dL [19].

**Figure 1:**
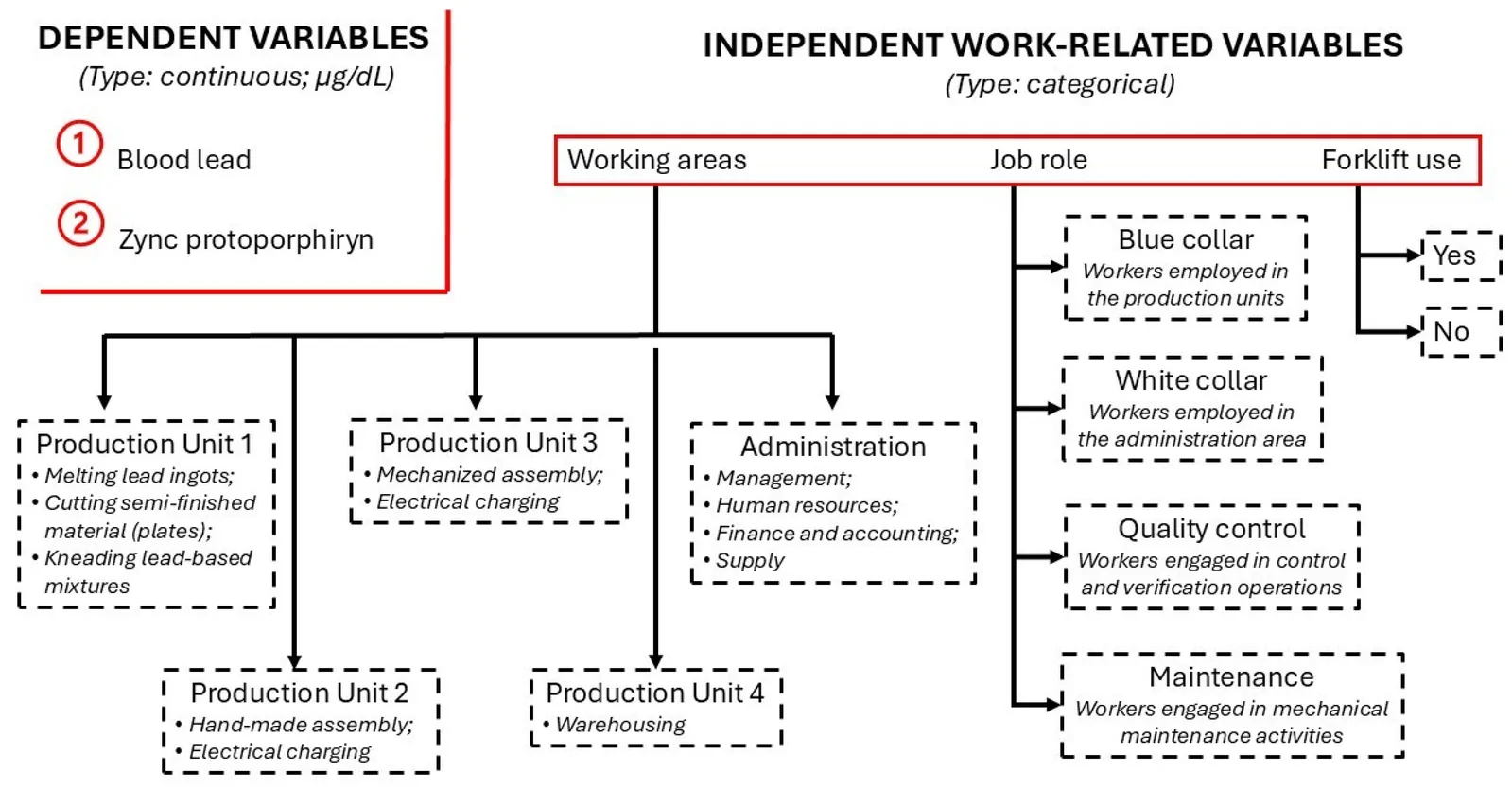
Study plan: Description of the exposure and effect biomarkers assessed and of the job activities and working areas in which the involved workers are employed. Information about the kind of variables is also provided.

Information about job roles (blue collar, white collar, quality control, maintenance), forklift use (yes/no) and working areas (production unit 1 (PU1), production unit 2 (PU2), production unit 3 (PU3), production unit 4 (PU4), administration) were collected with the aim of finding possible associations with the two biomarkers. These working areas all belong to different buildings. These variables describe occupational characteristics and were not direct measures of external lead exposure. No environmental or task-based lead measurements were available for this study. We provided the spatial representation of PU 1, PU 2 and PU 3 in the Supplementary Material (Figures S1–S3). PU 4 representation is not provided since it is a rectangular-plan building used as a warehouse. A detailed description of the cited occupational-related variables is provided in Figure 1.

More specifically, blue-collar workers only operate in the production units, and white-collar workers only operate in the administration area. Maintenance workers usually operate in all the production units, moving according to specific needs. Quality control workers usually operate in the administration area, but they spend at least part of the working shift in the production units.

The use of respiratory personal protective equipment (PPE) is mandatory for the exposed workers (blue collars, quality control, maintenance). Despite this, in the present study, no verification was performed of actual PPE compliance or its potential effect on the biological levels of the two markers evaluated.

Individual/clinical parameters such as age, seniority, gender, blood pressure (minimum and maximum blood pressure (mm/Hg) and a dichotomous variable called “hypertension” (over 130/80 mm/Hg, yes/no)), hemoglobin, triglycerides, cholesterol, blood glucose, body mass index (BMI), creatinine and medication intake (yes/no) were analyzed. Apart from hypertension and medication intake, the other variables are continuous. Lifestyle/hygienic information such as smoking (yes/no), cigarettes per day (continuous), years of smoking (continuous), alcohol consumption (both continuous (units per day) and categorical (yes/no)), physical activity (both continuous (hours per week) and categorical (yes/no)), nail-biting (yes/no), having a beard (yes/no, based on shaving at least every two days) and long hair (yes/no, based on the hair reaching the jawline), eating in the plant dining hall (yes/no) and using the worksite shower (yes/no) were also analyzed. Lifestyle/hygienic information was collected through voluntary informal interviews.

Statistical analyses were performed to explore the relationship between blood lead (PbB) and zinc protoporphyrin (ZnPPB) levels and occupational, individual/clinical, and lifestyle/hygienic characteristics. Continuous variables were summarized using mean and standard deviation (SD) or median and interquartile range (IQR), while categorical variables were described using absolute frequencies and percentages. The distribution of PbB and ZnPPB values was evaluated descriptively.

Differences in PbB and ZnPP levels across occupational categories, including job role and working area, were assessed using the Kruskal–Wallis test. When statistically significant differences were detected, post hoc pairwise comparisons were performed using the Dwass–Steel–Critchlow–Fligner test. Differences in PbB and ZnPPB levels according to dichotomous occupational, individual/clinical, or lifestyle/hygienic variables were assessed using the Mann–Whitney U test.

The relationships between PbB and ZnPPB levels and quantitative individual/clinical or lifestyle variables were assessed using Spearman’s rank correlation coefficient. The following continuous variables were included in the correlation analysis: age (years), seniority (years), cigarettes per day (n), years of smoking (years), alcohol consumption (units per day), physical activity (hours per week), maximum blood pressure (mm/Hg), minimum blood pressure (mm/Hg), body mass index, hemoglobin (mg/dL), triglycerides (mg/dL), cholesterol (mg/dL), blood glucose (mg/dL), and creatinine (mg/dL). When statistically significant differences were detected, post hoc pairwise comparisons were performed using the Dwass–Steel–Critchlow–Fligner (DSCF) test, which inherently adjusts *p*-values for multiple pairwise comparisons by controlling the family-wise error rate.

To further investigate the associations observed in the univariate analyses, multiple linear regression models were fitted using PbB and ZnPPB as dependent variables. Independent variables were selected based on statistical significance in the univariate analyses and on their clinical or occupational relevance. For PbB, occupational exposure-related variables were considered as potential confounders and were included when appropriate to account for differences in exposure profiles. Regression coefficients, 95% confidence intervals, and *p*-values were reported. To address potential overlap, we conducted a formal collinearity assessment within an extended multivariable linear regression model incorporating both individual traits and all occupational variables (gender, long hair, smoking, forklift use, job role, and working area). The diagnostic metrics confirmed the absence of severe multicollinearity, with Variance Inflation Factors (VIFs) remaining well below 2.0 for all predictors (ranging from 1.02 for long hair to 1.51 for job role; Tolerance > 0.66). 

The relationship between PbB and ZnPP levels was assessed using Spearman’s rank correlation coefficient. A scatterplot was used to graphically display the association between the two biomarkers.

A two-sided *p*-value < 0.05 was considered statistically significant. Statistical analyses were conducted using Jamovi software version 2.6 for Windows. Due to the exploratory nature of the study, no single primary outcome was pre-specified. To control the inflation of Type I error resulting from multiple hypothesis testing across univariate comparisons, the Benjamini–Hochberg False Discovery Rate (FDR) procedure was applied. Adjusted *p*-values (q-values) < 0.05 were considered statistically significant.

## 3. Results

Descriptive information about quantitative individual/clinical and lifestyle/hygienic variables and categorical variables is reported in Table 1. Continuous data pertain to age, seniority, cigarettes per day, years of smoking, alcohol consumption, physical activity, maximal blood pressure, minimal blood pressure, body mass index, and levels of hemoglobin, triglycerides, cholesterol, blood glucose and creatinine. Categorical information is related to gender, blood pressure (hypertension), smoking, medication intake, alcohol consumption and physical activity, worksite shower use, onychophagia, use of the dining hall service, having a long beard, and long hair.

Lead and ZnPP blood values in workers employed in the same factory were assessed. Workers were engaged in different job tasks and working areas. Forklift use was a work-related feature common to several workers (309) assigned to different production areas. PbB concentrations differed across job roles and working areas in the unadjusted analyses. No statistically significant differences in ZnPP concentrations were observed across these categories with regard to “Forklift use”; we tested possible differences with the non-parametric Mann–Whitney U test. Once again, we found a significant association with PbB levels but not with ZnPP values. Table 2 summarizes these findings.

Post hoc pairwise comparisons using the DSCF test were performed to identify which occupational groups differed significantly in PbB levels. Table 3 only reports statistically significant associations found through the DSCF test. The comparison between the totality of the tested variables is provided in the Supplementary Material (Table S1). The pairwise comparison of ZnPP values did not show significant differences for “Job role” and “Working area”.

A possible impact of lifestyle/hygienic features on the exposure (lead) and on the effect biomarker (ZnPP) blood levels was estimated. As highlighted in Table 4, PbB values are significantly associated with gender, smoking habits and long hair. Other variables appear not to be associated with PbB levels. Zinc protoporphyrin blood values, instead, appear to be associated with hypertension and physical activity. These associations are partially confirmed after *p*-value adjustment. After stratifying for job role (blue collar, maintenance) and working areas (production units 1, 2, 3 and 4), no significant differences were observed regarding hygienic features such as “use of the dining hall”, “long hair”, “onychophagia” and “use of the worksite shower” in lead and ZnPP blood values.

Table 5 shows the multivariable linear regression models for PbB (R2 = 0.066) and ZnPP (R2 = 0.037). In the adjusted models, gender (*p* = 0.007) and long hair (*p* = 0.009) retained statistically significant, though modest, independent associations with PbB levels, whereas smoking was no longer significant. For ZnPP, physical activity (*p* = 0.009) and hypertension (*p* = 0.013) retained independent inverse and direct associations, respectively. Overall, these models accounted for a small fraction of the total biomarker variance.

**Figure 2:**
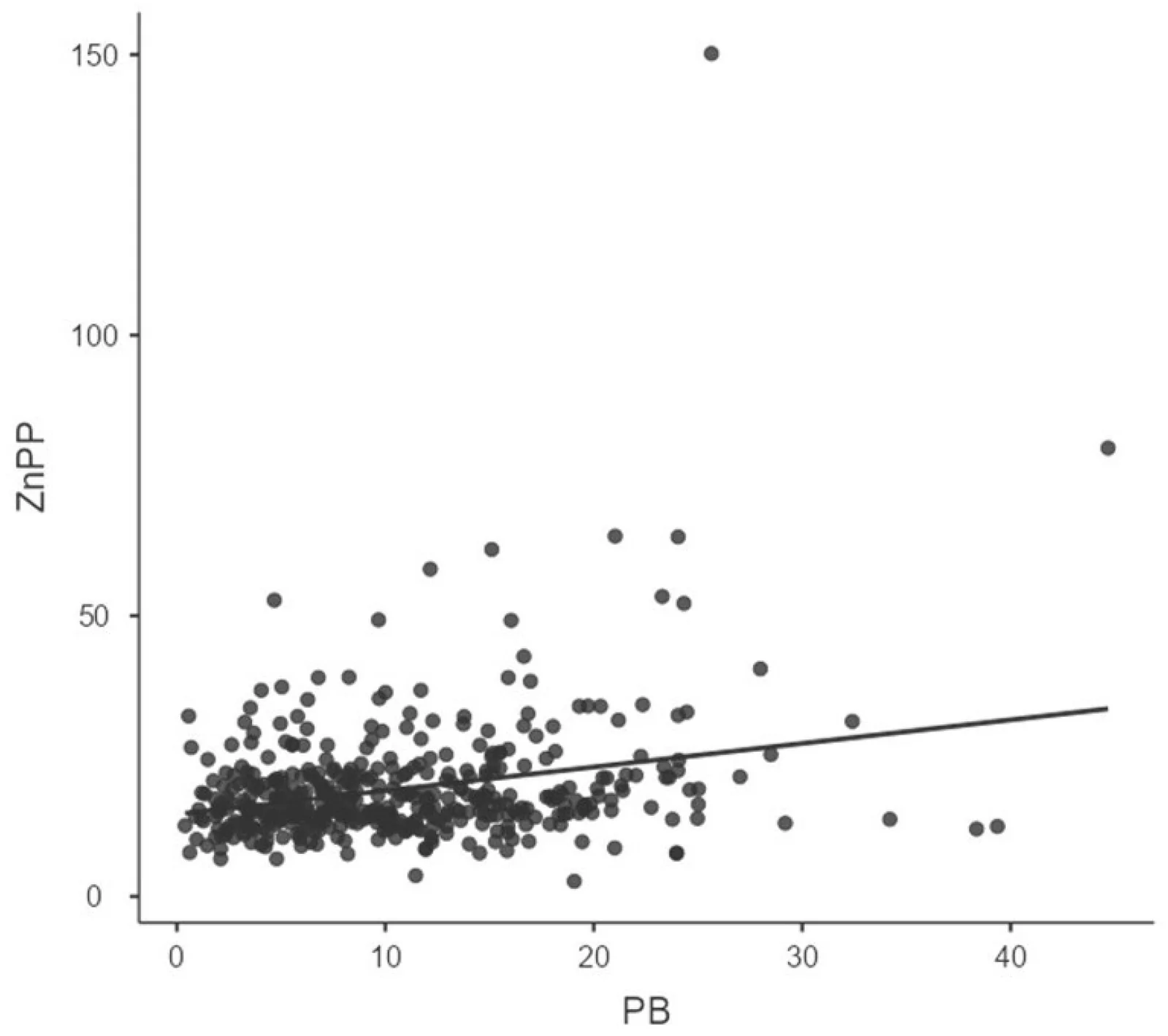
Scatter plot representing the relationship between ZnPPB (*y*-axis) and PbB (*x*-axis) in the whole sample. Values for both biomarkers are expressed as µg/dL.

Spearman’s rank correlation analysis was performed in relation to quantitative variables and PbB and ZnPP values. In the overall sample, blood lead (PbB) and ZnPP levels showed a weak but statistically significant positive correlation (rho = 0.176, *p* < 0.001). A sensitivity analysis performed after excluding the extreme ZnPP outlier (>150 µg/dL) confirmed that the relationship remained statistically significant (rho = 0.171, *p* = 0.003), demonstrating that the observed association was not driven by extreme observations. Figure 2 presents the scatterplot representing the relationship between the two biomarkers.

## 4. Discussion

Blood lead levels differed significantly according to both job role and working area. In particular, blue-collar and maintenance workers had higher mean PbB levels compared with white-collar workers, while production unit workers exhibited higher PbB levels than administrative staff. These findings are in line with prior occupational studies showing that workers in lead-related industrial environments have higher PbB concentrations compared with those in non-lead work roles (e.g., battery manufacturing, metal processing) [20,21]. Occupational exposure to inorganic lead is known to occur primarily via inhalation of lead-containing dust and fume and ingestion of contaminated particles, especially in settings with poor control measures, and is a common risk in manufacturing and industrial workplaces [22]. Globally, lead exposure remains an important occupational health concern: working with lead-containing products or in environments where lead dust is generated continues to be a risk for a diverse array of workers, such as smelters, battery production and recycling staff, construction workers and painters, emphasizing the need for vigilance in industrial hygiene [23].

Forklift use was associated with higher PbB concentrations in the unadjusted comparison. However, without environmental or task-based measurements, this finding cannot be attributed to a specific exposure mechanism. Forklift use may also reflect differences in working area, job activities, or other unmeasured occupational characteristics. Dust resuspension is a possible explanation, but it was not investigated in this study. While specific peer-reviewed studies on forklift operation and lead exposure are not available, which makes it difficult to contextualize our findings, the scientific literature [24] emphasizes that vehicle use may strongly influence the resuspension and the concentration of dust in air. Factors such as vehicle speed and weight may increase the average concentration of total and respirable dust. Conversely, water content in dust may significantly reduce dust resuspension. Exposures to toxic metals, including lead, can occur in a wide variety of industrial processes where dust concentrations are high, or ventilation is inadequate [25]. Although precise mechanisms linking forklift operation to increased lead exposure require further research, it is plausible that forklift use contributes to resuspension of settled lead-containing particulates, resulting in higher inhalation exposure with respect to workers not employed in forklift use.

In contrast to PbB, ZnPP values did not vary significantly across job roles, working areas, or forklift use. This observation aligns with the known biological behavior of ZnPP: it is a biomarker of effect used for detecting iron deficiency and reflecting disruption in heme synthesis, often with a lag relative to changes in PbB levels, and may not accurately capture short-term or moderate fluctuations in exposure typical of certain occupational tasks [13]. Despite this, as shown in Table 2, it should be noted that workers engaged in production units 1, 2 and 3 exhibited higher blood ZnPP values with respect to those engaged in the administration or in PU 4 (the warehouse). Previous research showed correlations between PbB and ZnPP, but also highlighted that ZnPP can be less sensitive than PbB for detecting lower or intermediate exposure levels in occupational settings [26]. Contrary to what we highlighted, Roh et al. [27] identified significant variations between ZnPP values in two groups of workers. Exposed workers showed higher PbB levels and ZnPP levels with respect to non-exposed workers. It should be pointed out, however, that the exposure values they found were much higher than ours, both for the exposed group (more than 40 µg/dL PbB) and for the non-exposed one (more than 10 µg/dL PbB). The observed difference could be due to a worse toxicological occupational-related condition.

The analysis of individual and lifestyle/hygienic variables provided interesting insights. Blood lead levels were significantly associated with gender, smoking habits, and hair length in the univariate analysis. The higher PbB concentrations observed in male workers are consistent with broader exposure data showing that occupational lead exposure disproportionately affects male workers in industries historically associated with higher hazard burdens [28]. To ensure a proper interpretation of this finding, it should be noted that, in the present study sample, female workers belong predominantly to the white-collar group. The association with smoking could relate to additional lead ingestion or hand-to-mouth transfer of contaminants. However, this association was not confirmed in the multivariate regression, suggesting it may be confounded by job-related factors. According to the Centers for Disease Control and Prevention’s NIOSH, hygienic and behavioral actions on the worksite may reduce lead intake [29]: high-frequency hand washing before eating, drinking and smoking is crucial. Moreover, heavy metal presence in cigarette smoke is a widely recognized public health problem [30], and smokers were found to have higher PbB levels with respect to non-smokers [31]. With regard to hair, it is known that it is a reliable matrix for lead biomonitoring, due to its ability to absorb the metal in the keratinous matrix [32]. Significant correlations between hair length and PbB levels were found [33]. Due to what is stated so far, we analyzed the relationship between hair length and blood lead and ZnPP values to assess whether longer hair might influence the amount of internal lead. Our findings highlight that longer hair is associated with lower PbB levels. To the best of our knowledge, no pertinent scientific references explain such an association. We hypothesize that longer hair requires more frequent hair care, and this reduces the amount of lead contamination. Such an interesting revelation should deserve further research.

Other lifestyle/hygienic variables did not show significant associations with PbB levels. The use of the worksite shower, if available, is recommended for lead removal from skin and hair [29]. In our sample, this hygienic behavior did not appear linked to PbB levels.

As already stated, similar advice is given with respect to hand-washing. Due to this, we would expect a clearer difference in PbB levels between nail-biting workers and non-nail-biting ones. Despite the absence of statistical significance, a difference was observed (13.3 µg/dL in nail-biters vs 11.33 µg/dL in non-nail-biters). According to the World Health Organization (WHO), hand-to-mouth activities are associated with higher PbB levels in humans (e.g., in children) [34]. Moreover, oral intake of inorganic lead may be influenced by eating in non-separated areas and by hand-to-mouth unhygienic behaviors [29,35]. Due to this, we looked for differences between workers who declared they use the workplace dining hall and those who did not. We detected a slight and non-statistically significant difference (11.4 µg/dL for dining hall users and 12.3 µg/dL for non-users). This outcome may be linked to good hygienic practices in the dining hall and the absence of lead contamination from the polluted areas of the industrial plant.

A recent meta-analysis [36] involving more than 180.000 workers revealed a relationship between PbB levels and hypertension. More specifically, the authors highlighted that lead exposure increases the risk of hypertension and shows a clear dose–response relationship. In our setting, such an association was not confirmed.

Long facial hair may decrease respiratory PPE efficacy [37]. Moreover, facial skin contamination in lead-exposed workers is a recognized risk factor [35]. For these reasons, we investigated the relationship between PbB levels and beard length. No significant differences were observed.

Several non-chelating medications or supplements have been reported to influence PbB levels: ascorbic acid [38] or calcium-based supplements [39]. Although our investigation did not explore the kind of medication (it was limited to a binary indicator, yes/no), no differences were observed on a medication-intake basis.

Lifestyle factors can influence xenobiotic blood levels [40]. With regard to this aspect, we explored the relationship between smoking and physical activity and PbB levels. In our sample, such factors appear not to influence the amount of inorganic lead in the hematic matrix.

In the exploratory multivariable analysis, ZnPP concentrations were associated with hypertension and physical activity. However, the model explained only 3.65% of the variance, and these associations do not establish lead-related biological effects or mechanisms influencing ZnPP. Notably, our findings regarding blood pressure diverge from the previous literature: Italian researchers [41] as well as a large study of over 700 subjects [42] reported no link between ZnPP and blood pressure. Conversely, our observations align with Poreba et al. [43], who identified older age, elevated BMI, and higher ZnPP levels as significant predictors of hypertension.

With regard to the association between ZnPP values and physical activity in exposed workers, scientific evidence is sparse. A study by Staessen et al. [42] took into account physical activity and did not highlight significant associations. Scientific evidence mainly focuses on the study of ZnPP as involved in iron deficiency. Despite the difficulty in finding comparable studies, our results show a clear difference in ZnPP levels between workers who declare that they exercise and those who do not; for both groups, the ZnPP values fall within the reference range [44]. Lower ZnPP concentrations were observed among workers reporting physical activity. The biological and clinical significance of this difference remains uncertain.

No further significant associations were found between blood ZnPP levels and lifestyle/hygienic variables, despite a slight difference being observed in ZnPP blood values between nail-biters and non-nail-biters. The second group showed higher levels.

PbB and ZnPP showed a weak positive correlation. This association alone does not establish that the observed ZnPP concentrations reflect lead-related disruption of heme synthesis in this population. Blood lead primarily reflects recent exposure, whereas ZnPP reflects cumulative disruption in heme synthesis pathways over longer time frames. Nevertheless, other researchers demonstrated that PbB and ZnPP correlation is strong when lead intoxication is relevant; no correlation was found among workers with low PbB and ZnPP levels [27]. In this case, PbB levels, comparable to those found in our exposed workers (approximately 10 µg/dL), did not show a correlation with ZnPP. Such a finding is fully comparable to ours. Our observations, showing that ZnPP did not significantly vary by job role or working area despite differences in PbB, align with studies that highlight the complex relationship between PbB and ZnPP in occupational settings. Previous longitudinal research in battery factory workers reported a linear relationship between log-ZnPP and PbB across a wide range of exposure levels [45]. An investigation among Korean lead workers demonstrated that the association between corrected PbB and ZnPP is influenced by serum iron status, suggesting biological modifiers of the PbB–ZnPP relationship [46]. Similar occupational studies, including automobile workshop workers, also documented significantly elevated ZnPP values in lead-exposed workers compared to controls, supporting the use of ZnPP as a complementary biomarker in occupational surveillance. Regarding the relationship highlighted in the sample studied in this study (Figure 2), no previous study reported a similar trend [27,45,46]. Visual evaluation of the PbB–ZnPP relationship highlighted an extreme ZnPP outlier (~150 µg/dL). However, because Spearman’s rank correlation operates on ranks rather than raw values, and as confirmed by our sensitivity analysis excluding outliers, this extreme measurement did not artificially induce the observed statistical significance. The cited studies pointed out strong correlations and, globally, clear linear relationships between PbB and ZnPP. Once again, it must be emphasized that exposure levels described by other researchers [27,45,46] are two- to four-fold higher than those measured in our sample. Such an aspect may explain the differences between our results and those of others; given the low blood lead levels characteristic of our study population, a weak association between the two biomarkers is predictable.

The correlation analysis (Table S2) highlighted several significant but weak correlations, both for PbB and blood ZnPP values. Although it did not focus on correlation analysis, the study by Roh et al. [27] demonstrated that both PbB and ZnPP levels increase as age increases. Similar findings are reported in a recent study by Mohammed et al. [47], with, in addition, an increased risk of higher PbB levels among workers with longer employment duration. Such a characteristic is confirmed by the significant correlation that we found between seniority and PbB levels. Similarly to the results of this study, Santos et al. [18] found weak correlations between ZnPP blood levels and age and seniority but, in their analysis, no statistical significance was highlighted.

The present study highlighted a significant weak correlation between PbB levels and smoking. The number of cigarettes used per day and the number of years of smoking were quantified. A recent study assessing lung cancer risk in Korean workers [31] found that the proportion of current smokers was preponderant in the group with the highest blood Pb levels. These findings are consistent with our results.

Data collected in this study reported a significant positive correlation between PbB levels and hemoglobin (Rho = 0.189, *p* < 0.001). This finding is in contrast with the existing literature. The study by Counter et al. [48] highlighted, despite being non-significant, a negative correlation. The relationship between lead exposure and anemia is well established [13,42], but some authors reported that this association is detectable for PbB levels higher than 40 µg/dL [49]. In the sample of workers involved in our analysis, average PbB levels were overall lower than this value.

Regarding physical activity, the identified significant negative correlation is consistent with the finding that was already discussed: in the sample studied, as the number of hours of physical activity increases, ZnPP levels decrease. As reported by Yang et al. [36], this protective effect in lead-exposed workers may be due to the physical activity enhancement of the body’s antioxidant defense capacity, thereby offsetting lead-induced oxidative stress.

A positive and significant weak correlation between ZnPP values and max blood pressure was identified. This finding is consistent with the associations observed between ZnPP and hypertension, also reported in a previous study, as already discussed [43].

With regard to BMI, peer-reviewed studies assessing the relationship between blood ZnPP and body mass index do not seem to be available. Despite this, an indirect evaluation is possible. A study published in 2022 [49] compared ZnPP values in high- and low-exposed groups: ZnPP levels were found to be significantly different between groups, while BMI was not. A study by Wang et al. [50] conducted in China reported robust evidence of an association between BMI and PbB levels. Unfortunately, the ZnPP values were not assessed as an effect biomarker of lead exposure. Considering that lead accumulates mainly in blood and bones [2], not in fat, the significant correlation found between BMI and a marker of chronic lead exposure like ZnPP should deserve further investigation.

Finally, regarding cholesterol, our findings align with those of Poreba et al. [51]. The authors found that lead-exposed workers showed statistically significant total cholesterol concentration with respect to non-exposed workers, along with higher blood pressure levels. Individuals exposed to lead compounds, compared with non-exposed individuals, also showed significantly higher mean concentrations of LDL cholesterol. Conversely, a less recent study conducted in India [52] found that ZnPP blood values were not associated with differences between exposed and control groups.

The limitations of this study include its cross-sectional design, which limits inference about causality, and the lack of prior literature on forklift operation specifically as a lead exposure risk factor and on clear relationships between ZnPP and hygienic/behavioral practices. The relatively small size of some subgroups (e.g., quality control staff and female workers) may also have reduced statistical power for some comparisons. The absence of environmental and task-based lead measurements prevents attribution of the observed PbB differences to specific workplace sources or exposure routes. Occupational characteristics cannot therefore be interpreted as established exposure determinants, and residual occupational confounding remains possible. A key methodological limitation is the low coefficient of determination observed in our regression models. In cross-sectional biomonitoring studies of populations with low-level occupational exposure, a large proportion of biomarker variance is routinely driven by unmeasured inter-individual, dietary, metabolic, and environmental factors. Consequently, the statistically significant regression coefficients reported here reflect weak-to-modest independent tendencies rather than strong predictive or causal relationships. Another limitation relates to the exploratory nature of our study design and the potential for inflated Type I error due to multiple hypothesis testing across numerous occupational, clinical, and lifestyle variables. While we controlled the FDR using the Benjamini–Hochberg procedure for univariate analyses, findings—particularly regarding novel behavioral variables such as hair length or specific hygienic practices—should be interpreted as hypothesis-generating. Future prospective studies with pre-specified primary hypotheses and targeted exposure measurements are warranted to confirm these observed associations. Finally, most information collection was based on voluntary participation; this led to several instances of missing data during data retrieval.

Iron status was not assessed, limiting the interpretation of ZnPP concentrations and their associations with individual characteristics. Moreover, this study did not formally evaluate the diagnostic performance or clinical utility of ZnPP as a biomarker of lead-related effects. The study did not include repeated biomarker measurements or a dedicated assessment of analytical imprecision and within-person biological variability. We therefore cannot quantify their contribution to the observed variation or establish whether the exploratory associations are reproducible. Statistical significance alone should not be interpreted as evidence of biological or clinical relevance.

## 5. Conclusions

PbB concentrations differed across job roles and working areas, and an unadjusted difference was also observed according to forklift use. These findings do not establish causal effects or identify the sources and mechanisms responsible for the observed differences. ZnPP showed a weak positive correlation with PbB and no statistically significant differences across the occupational categories examined. The associations observed with hypertension and physical activity remain exploratory and should not be interpreted as evidence of lead-related biological effects. This study did not formally evaluate the diagnostic performance or clinical utility of ZnPP and therefore cannot establish its suitability as an effect biomarker in this setting. Further studies incorporating iron status assessment and environmental exposure measurements are needed to clarify the interpretation of these findings.

Both for some individual/clinical parameters and hygienic parameters analyzed in this study, the previous scientific literature is sparse. For this reason, our results offer interesting insights for consideration of these factors in future and specific studies. The results could be used to improve the working conditions of exposed workers and to better define the contents of occupational safety and hygienic behavioral training, with the aim of reducing and preventing lead exposure and the related effects. These observations may inform the selection of hypotheses for future studies combining biomonitoring with environmental and task-based exposure assessment.

## Figures and Tables

**Table 1:** Descriptive information about quantitative individual/clinical and lifestyle/hygienic variables and about categorical lifestyle/hygienic data. Continuous variables are presented as mean, standard deviation or median and interquartile range. Crude frequencies and relative percentages are provided for categorical variables.

Variable		Observations	Median (IQR)		M (SD)	
Age (years)		464	44.00 (36.00–56.00)		45.27 (11.72)	
Seniority (years)		464	10.00 (4.00–27.00)		14.29 (12.16)	
Cigarettes per day (N)		225	0 (0–10.00)		6.47 (7.95)	
Years of smoking (years)		225	0 (0–20.00)		9.7 (11.5)	
Alcohol (units per day)		47	0 (0–0)		0.11 (0.32)	
Physical activity (hours per week)		457	2 (0–3.00)		2.14 (2.34)	
Max blood pressure (mm/Hg)		461	120 (120.00–130.00)		125.3 (11.75)	
Min blood pressure (mm/Hg)		461	80 (70.00–80.00)		78.01 (8.01)	
Body Mass Index (N)		462	26.10 (23.8–28.4)		26.36 (3.64)	
Hemoglobin (mg/dL)		447	15.40 (14.7–16.00)		15.32 (1.07)	
Triglycerides (mg/dL)		436	132 (92.92–193.25)		166.45 (123.25)	
Cholesterol (mg/dL)		447	192 (165.00–219.00)		195.76 (84.23)	
Blood glucose (mg/dL)		447	85.30 (78.8–94.45)		89.30 (23.88)	
Creatinine (mg/dL)		447	0.94 (0.86–1.04)		0.95 (0.15)	
Variable	Frequency (N)	Relative percentage %	Variable	Frequency (N)		Relative percentage %
Gender			Beard			
Male	450	97.0	Yes	113		39.2
Female	14	3.0	No	175		60.8
Hypertension			Long hair			
Yes	69	15.2	Yes	114		43.2
No	384	84.8	No	150		56.8
Smoking			Nail-biting			
Yes	225	49.2	Yes	41		15.5
No	232	50.8	No	223		84.5
Alcohol			Worksite shower			
Yes	47	10.3	Yes	230		92.7
No	409	89.7	No	18		7.3
Medication			Physical activity			
Yes	153	33.3	Yes	269		58.9
No	306	66.7	No	188		41.1
Dining hall						
Yes	216	80.3				
No	53	19.7				

**Table 2:** Descriptive and inferential information about PbB and ZnPP values in the enrolled workers. Variables are shown on the basis of occupational characteristics such as “Job role”, “Working area” and “Forklift use”. Values are expressed as µg/dL; standard deviation and a statistical analysis of possible differences are also provided. Statistical significance is highlighted in bold. * Kruskal–Wallis test; ** Mann–Whitney U test. PU = Production unit.

Job Role (N)	Median Blood Lead (µg/dL) (IQR)	Mean Blood Lead (µg/dL) (SD)	*p*-Value
blue collar (401)	9.71 (5.86–15.14)	11.11 (6.87)	<0.001 *
white collar (37)	3.18 (1.99–5.62)	4.69 (4.92)	
quality control (6)	10.80 (5.01–13.14)	9.41 (6.26)	
maintenance (20)	10.29 (5.90–14.95)	10.42 (5.32)	
Job role (N)	Median blood ZnPP (µg/dL) (IQR)	Mean blood ZnPP (µg/dL) (SD)	*p*-value
blue collar (389)	16.32 (13.88–21.67)	19.43 (11.46)	0.594 *
white collar (35)	16.94 (13.48–21.71)	16.51 (6.70)	
quality control (6)	16.86 (14.49–19.09)	17.98 (4.74)	
maintenance (20)	18.94 (13.48–21.71)	18.81 (6.70)	
Working areas (N)	Median blood lead (µg/dL) (IQR)	Mean blood lead (µg/dL) (SD)	*p*-value
Production-PU 1 (144)	11.25 (7.21–17.04)	12.67 (7.14)	<0.001 *
Production-PU 2 (185)	10.38 (6.06–15.49)	11.18 (6.46)	
Production-PU 3 (62)	6.94 (4.66–11.92)	8.99 (7.02)	
Production-PU 4 (33)	7.06 (4.99–8.97)	7.40 (3.50)	
Administration (40)	3.29 (1.95–5.71)	4.93 (5.22)	
Working areas (N)	Median blood ZnPP (µg/dL) (IQR)	Mean blood ZnPP (µg/dL) (SD)	*p*-value
Production-PU 1 (141)	17.17 (14.30–21.67)	19.78 (13.54)	0.064 *
Production-PU 2 (180)	16.30 (13.68–21.42)	19.12 (9.29)	
Production-PU 3 (60)	17.41 (14.49–22.57)	21.15 (12.58)	
Production-PU 4 (31)	14.79 (12.89–17.98)	15.76 (5.07)	
Administration (38)	16.52 (12.77–19.19)	16.60 (5.72)	
Forklift use (N)	Median blood lead (µg/dL) (IQR)	Mean blood lead (µg/dL) (SD)	*p*-value
Yes (310)	9.95 (5.66–15.1)	10.93 (6.62)	0.018 **
No (153)	8.15 (4.65–13.0)	9.80 (7.32)	
Forklift use (N)	Median blood ZnPP (µg/dL) (IQR)	Mean blood ZnPP (µg/dL) (SD)	*p*-value
Yes (299)	16.28 (13.53–21.5)	19.01 (11.35)	0.467 **
No (150)	17.19 (14.13–21.4)	19.45 (9.99)	

**Table 3:** Pairwise comparison between statistically significant PbB values with regard to ‘Job roles’ and ‘Working areas’. PU = Production unit. Note: *p*-values were obtained via the Dwass–Steel–Critchlow–Fligner (DSCF) post hoc test and are inherently adjusted for multiple pairwise comparisons.

Job Role			
Pairwise Comparison-Blood Lead		Statistics	*p*-Value
Blue collar	White collar	−9.1153	<0.001
Maintenance	White collar	−5.6591	<0.001
Working areas			
Pairwise comparison-Blood Lead		Statistics	*p*-value
Production-PU 2	Production-PU 4	−4.591	0.010
Production-PU 2	Production-PU 3	−4.128	0.029
Production-PU 2	Administration	−8.674	<0.001
Production-PU 4	Production-PU 1	5.784	<0.001
Production-PU 4	Administration	−5.370	<0.001
Production-PU 1	Production-PU 3	−5.675	<0.001
Production-PU 1	Administration	−9.427	<0.001
Production-PU 3	Administration	−5.899	<0.001

**Table 4:** Comparison of PbB and ZnPP values in association with lifestyle/hygienic variables. Statistical significance was assessed using the Mann–Whitney U test. *p*-values were adjusted for multiple testing using the Benjamini–Hochberg False Discovery Rate (FDR) procedure.

Lifestyle/Hygienic				
Blood Lead				
Variable (N)	Median Value in Blood (µg/dL) (IQR)	Mean Value in Blood (µg/dL) (SD)	*p*-Value	FDR Adj *p*-Value
Male (450)	9.61 (5.55–14.91)	10.8 (6.79)	<0.001	<0.001
Female (14)	1.54 (0.962–3.03)	3.64 (6.10)		
Hypertension-yes (69)	8.97 (5.41–14.9)	11.0 (7.25)	0.727	0.787
Hypertension-no (384)	9.34 (5.39–14.8)	10.5 (6.82)		
Smoking-yes (225)	9.86 (6.04–15.3)	11.3 (7.17)	0.028	0.103
Smoking-no (232)	8.41 (4.76–14.4)	9.90 (6.48)		
Alcohol-yes (47)	9.69 (5.70–12.6)	10.3 (5.51)	0.888	0.888
Alcohol-no (409)	9.31 (5.41–14.9)	10.6 (7.00)		
Medication-yes (153)	8.59 (4.97–14.6)	10.2 (6.68)	0.385	0.638
Medication-no (306)	9.68 (5.55–14.9)	10.8 (6.97)		
Dining hall-yes (216)	10.2 (5.95–15.3)	11.4 (7.21)	0.423	0.638
Dining hall-no (53)	11.4 (5.79–17.7)	12.3 (7.69)		
Beard-yes (113)	10.25 (6.32–16.0)	11.7 (7.55)	0.560	0.722
Beard-no (175)	9.94 (5.44–15.3)	11.2 (7.00)		
Long hair-yes (114)	8.91 (5.25–14.5)	9.94 (6.29)	0.002	0.011
Long hair-no (150)	11.94 (6.91–17.1)	12.9 (7.80)		
Nail-biting-yes (41)	12.9 (7.24–18.3)	13.3 (7.33)	0.078	0.215
Nail-biting-no (223)	10.1 (5.62–15.4)	11.3 (7.31)		
Worksite shower-yes (230)	10.32 (5.99–15.7)	11.7 (7.21)	0.203	0.447
Worksite shower-no (18)	6.52 (3.85–16.7)	10.7 (10.10)		
Physical activity-yes (269)	9.61 (5.55–14.8)	10.7 (7.02)	0.715	0.787
Physical activity-no (188)	9.15 (5.22–14.9)	10.4 (6.60)		
Zinc protoporphyrin				
Variable (N)	Median value in blood (µg/dL) (IQR)	Mean value in blood (µg/dL) (SD)	*p*-value	FDR Adj *p*-value
Male (437)	16.43 (13.79–21.44)	19.24 (10.98)	0.282	0.341
Female (13)	16.09 (10.16–18.26)	16.24 (7.25)		
Hypertension-yes (66)	18.6 (14.9–24.8)	23.0 (8.83)	0.014	0.077
Hypertension-no (373)	16.1 (13.6–21.2)	18.5 (18.6		
Smoking-yes (217)	17.1 (14.2–21.9)	19.4 (12.29)	0.401	0.697
Smoking-no (226)	16.1 (13.5–21.2)	18.98 (9.52)		
Alcohol-yes (46)	15.8 (13.8–21.8)	18.4 (5.73)	0.824	0.824
Alcohol-no (396)	16.4 (13.7–21.4)	19.3 (11.43)		
Medication-yes (146)	17.1 (14.5–22.4)	20.4 (13.87)	0.127	0.349
Medication-no (299)	16.3 (13.5–21.2)	18.6 (9.14)		
Dining hall-yes (212)	16.8 (13.9–21.2)	19.1 (8.96)	0.642	0.852
Dining hall-no (51)	15.5 (13.4–22.3)	22.0 (21.76)		
Beard-yes (111)	16.4 (13.7–22.3)	20.3 (11.54)	0.645	0.852
Beard-no (170)	16.5 (13.8–21.3)	19.2 (12.65)		
Long hair-yes (111)	17.2 (14.1–21.5)	18.94 (8.32)	0.620	0.852
Long hair-no (148)	16.1 (13.6–21.3)	20.3 (14.94)		
Nail-biting-yes (41)	18.3 (14.51–25.9)	21.4 (10.95)	0.179	0.394
Nail-biting-no (218)	16.4 (13.69–21.2)	19.4 (12.82)		
Worksite shower-yes (226)	16.42 (13.69–21.4)	19.4 (9.94)	0.426	0.697
Worksite shower-no (18)	19.29 (14.8–22.0)	26.4 (31.7)		
Physical activity-yes (262)	15.63 (13.38–20.1)	17.8 (8.51)	<0.001	0.006
Physical activity-no (181)	17.72 (14.68–23.1)	21.2 (13.54)		

**Table 5:** Multiple linear regression models to assess the association between the independent variables smoke, long hair and gender with PbB values (R2 = 0.0664) and the variables physical activity and hypertension with zinc protoporphyrin blood values (R2 = 0.0365).

Blood Lead Values				
Variable	Coefficient	Confidence Interval 95%		*p*-Value
Smoking				
Yes	Rif.			
No	1.47	−0.297	3.234	0.112
Long hair				
Yes	Rif.			
No	−2.33	−4.117	−0.543	0.009
Gender				
Female	Rif.			
Male	−6.09	−11.163	−1.018	0.007
Blood ZnPP values				
Variable	Coefficient	Confidence Interval 95%		*p*-value
Physical activity				
Yes	Rif.			
No	−2.82	−4.943	−0.693	0.009
Hypertension				
Yes	Rif.			
No	3.67	0.764	6.586	0.013

## Data Availability

The dataset supporting the conclusions of this article is available in the Zenodo repository, https://doi.org/10.5281/zenodo.21279621.

## Supplementary Materials

The following supporting information can be downloaded at https://www.mdpi.com/article/10.3390/toxics14100922/s1, Figure S1: Spatial representation of Production Unit 1, where 3 activities are performed: kneading, cutting and melting; Figure S2: Spatial representation of Production Unit 2, where 2 activities are performed: electrical charging and hand-made assembling; Figure S3: Spatial representation of Production Unit 3, where 2 activities are performed: electrical charging and mechanized assembling; Table S1: Pairwise comparison of blood lead and blood ZnPP values with respect to Job role and Working areas. Bold values represent statistically significant differences; Table S2: Spearman’s rank correlation analysis of blood lead and ZnPP values with continuous variables. Bold values represent statistically significant correlation.

## Abbreviations

The following abbreviations are used in this manuscript:

ZnPP	Zinc-protoporphyrin
ZnPPB	Blood zinc-protoporphyrin
Pb	Lead
PbB	Blood lead
ICP–MS	Inductively Coupled Plasma–Mass Spectrometry
HPLC-FL	High-Performance Liquid Chromatography coupled with Fluorescence Detection
PU	Production unit
PPE	Personal protective equipment
BMI	Body Mass Index
DSCF	Dwass–Steel–Critchlow–Fligner
WHO	World Health Organization
