# Supplementary material for: Workers’ Behavior and Hygienic Parameters Related to Blood Lead Levels: A Cross-Sectional Biomonitoring Study in an Italian Automotive Plant
Source: Toxics. 2026 Oct 18;14(10):922. doi: 10.3390/toxics14100922 (PMC13709819; doi:10.3390/toxics14100922)
Supplement: Supplementary file 1 [file toxics-14-00922-s001.zip › SUPPLEMENTARY - TABLE S1.pdf]

**Job role**			
**Pairwise comparison – Blood Lead**		**Statistics**	**p-value**
Blue collar	Maintenance	-0.0921	1000
Blue collar	White collar	-9.1153	**<0.001**
Blue collar	Quality control	-0.5552	0.978
Maintenance	White collar	-5.6591	**<0.001**
Maintenance	Quality control	-0.5164	0.983
White collar	Quality control	2.3385	0.315
**Pairwise comparison – ZnPP**		**Statistics**	**p-value**
Blue collar	Maintenance	0.613	0.976
Blue collar	White collar	-2.079	0.607
Blue collar	Quality control	0.184	0.999
Maintenance	White collar	-2.381	0.446
Maintenance	Quality control	-0.344	0.995
White collar	Quality control	1.125	0.911
**Working areas**			
**Pairwise comparison – Blood Lead**		**Statistics**	**p-value**
Production – PU 2	Production – PU 4	-4.591	**0.010**
Production – PU 2	Production – PU 1	2.306	0.418
Production – PU 2	Production – PU 3	-4.128	**0.029**
Production – PU 2	Administration	-8.674	**<.001**
Production – PU 4	Production – PU 1	5.784	**<.001**
Production – PU 4	Production – PU 3	0.464	0.998
Production – PU 4	Administration	-5.370	**<.001**
Production – PU 1	Production – PU 3	-5.675	**<.001**
Production – PU 1	Administration	-9.427	**<.001**
Production – PU 3	Administration	-5.899	**<.001**
**Pairwise comparison – ZnPP**		**Statistics**	**p-value**
Production – PU 2	Production – PU 4	-2.813	0.271
Production – PU 2	Production – PU 1	0.691	0.976
Production – PU 2	Production – PU 3	1.608	0.787
Production – PU 2	Administration	-2.100	0.721
Production – PU 4	Production – PU 1	3.269	0.127
Production – PU 4	Production – PU 3	3.547	0.089
Production – PU 4	Administration	0.801	0.947
Production – PU 1	Production – PU 3	1.135	0.951
Production – PU 1	Administration	-2.367	0.573
Production – PU 3	Administration	-2.820	0.384

Table S1 - Pairwise comparison of blood lead and blood ZnPP values with respect to Job role and Working areas. Bold values represent statistically significant differences
