# Supplementary material for: Workers’ Behavior and Hygienic Parameters Related to Blood Lead Levels: A Cross-Sectional Biomonitoring Study in an Italian Automotive Plant
Source: Toxics. 2026 Oct 18;14(10):922. doi: 10.3390/toxics14100922 (PMC13709819; doi:10.3390/toxics14100922)
Supplement: Supplementary file 1 [file toxics-14-00922-s001.zip › SUPPLEMENTARY - TABLE S2.pdf]

Quantitative variable	Blood lead values		Blood ZnPP values	
	Spearman's Rho	p-value	Spearman's Rho	p-value
Age (years)	0,125	**0,007**	0,135	**0,004**
Seniority (years)	0,134	**0,004**	0,093	**0,05**
Cigarettes per day (N)	0,121	**0,01**	0,076	0,109
Years of smoking (years)	0,120	**0,01**	0,051	0,28
Alcohol (units per day)	0,007	0,889	0,010	0,828
Physical activity (hours per week)	-0,006	0,898	-0,181	**<0,001**
Max blood pressure (mm/Hg)	0,054	0,25	0,151	**0,001**
Min blood pressure (mm/Hg)	0,005	0,92	0,05	0,28
Body Mass Index (N)	0,028	0,555	0,149	**0,002**
Hemoglobin (mg/dl)	0,189	**<0,001**	0,058	0,232
Triglycerides (mg/dl)	-0,028	0,563	0,04	0,415
Cholesterol (mg/dl)	0,038	0,418	0,109	**0,023**
Blood glucose (mg/dl)	0,041	0,387	0,072	0,135
Creatinine (mg/dl)	-0,010	0,826	-0,055	0,251

Table S2 - Spearman's rank correlation analysis of blood lead and ZnPP values with continuous variables. Bold values represent statistically significant correlation
